# SIRT6 is a key regulator of mitochondrial function in the brain

**DOI:** 10.1038/s41419-022-05542-w

**Published:** 2023-01-18

**Authors:** Dmitrii Smirnov, Ekaterina Eremenko, Daniel Stein, Shai Kaluski, Weronika Jasinska, Claudia Cosentino, Barbara Martinez-Pastor, Yariv Brotman, Raul Mostoslavsky, Ekaterina Khrameeva, Debra Toiber

**Affiliations:** 1 Department of Life Sciences, Ben-Gurion University of the Negev, Beer Sheva, 8410501 Israel; 2 The Zlotowski Center for Neuroscience, Ben-Gurion University of the Negev, Beer Sheva, 8410501 Israel; 3 Center for Molecular and Cellular Biology, Skolkovo Institute of Science and Technology, Moscow, 121205 Russia; 4 The Massachusetts General Hospital Cancer Center, Harvard Medical School, Boston, MA 02114 USA; 5 Molecular Oncology Program, Spanish National Cancer Research Center (CNIO), Madrid, 28029 Spain; 6 The Broad Institute of Harvard and MIT, Cambridge, MA 02142 USA

**Keywords:** Cell biology, Dementia, Molecular biology

## Abstract

The SIRT6 deacetylase has been implicated in DNA repair, telomere maintenance, glucose and lipid metabolism and, importantly, it has critical roles in the brain ranging from its development to neurodegeneration. Here, we combined transcriptomics and metabolomics approaches to characterize the functions of SIRT6 in mouse brains. Our analysis reveals that SIRT6 is a central regulator of mitochondrial activity in the brain. SIRT6 deficiency in the brain leads to mitochondrial deficiency with a global downregulation of mitochondria-related genes and pronounced changes in metabolite content. We suggest that SIRT6 affects mitochondrial functions through its interaction with the transcription factor YY1 that, together, regulate mitochondrial gene expression. Moreover, SIRT6 target genes include SIRT3 and SIRT4, which are significantly downregulated in SIRT6-deficient brains. Our results demonstrate that the lack of SIRT6 leads to decreased mitochondrial gene expression and metabolomic changes of TCA cycle byproducts, including increased ROS production, reduced mitochondrial number, and impaired membrane potential that can be partially rescued by restoring SIRT3 and SIRT4 levels. Importantly, the changes we observed in SIRT6-deficient brains are also occurring in aging human brains and particularly in patients with Alzheimer’s, Parkinson’s, Huntington’s, and Amyotrophic lateral sclerosis disease. Overall, our results suggest that the reduced levels of SIRT6 in the aging brain and neurodegeneration initiate mitochondrial dysfunction by altering gene expression, ROS production, and mitochondrial decay.

## Introduction

Aging is a consequence of the dysregulation of various self-maintenance mechanisms of a living system. Aging at the cellular level is accompanied by genomic instability, telomere shortening, loss of proteostasis, and mitochondrial dysfunction, together with a decrease in the efficiency of the DNA repair mechanism [1–3]. Moreover, these factors are interconnected. For example, the shortening of telomeres can lead to mitochondrial dysfunction [4]. Aging involves significant changes in the brain structure and functional capabilities [2, 5–7]. Cognitive decline occurs naturally during aging, but in some cases, it can become pathological, such as in neurodegenerative diseases. Importantly, about 95% of neurodegenerative cases are age-related with no known genetic mutation. Therefore, a better understanding of the aging process in disease development is needed.

Sirtuins are a family of proteins that have mono-ADP ribosyltransferase or deacetylase activity [8–10]. As one of the most notable proteins of this family, SIRT6 is implicated in genomic stability [11–14], DNA repair [13, 15], telomere maintenance [16], cellular metabolism [17] and importantly, it has critical roles in the protection against aging-associated diseases [18–20]. SIRT6-deficient mice have a progeroid (“premature aging-like”) syndrome with low body weight and a very short lifespan of ~four weeks compared to normal mice [11]. SIRT6 is widely expressed in mammalian brain tissues, with the highest expression level in cortical and hippocampal regions [21]. SIRT6 plays a neuroprotective role, protecting against DNA damage accumulation and during ischemic brain injury [18, 22]. The lack of SIRT6, specifically in the brain, results in learning and memory impairments, increased DNA damage, and the promotion of cortical apoptotic cells, partially through the hyperphosphorylation and hyperacetylation of Tau [18, 23]. In addition, through the changes in gene expression in these brains, we identified signatures of pathological aging, particularly relevant for A.D. and P.D., that could be partially reversed by calorie restriction [24]. Importantly, SIRT6 levels are decreased in the aging brains [18] and even more pronounced in Alzheimer’s patients [23], suggesting its involvement in age-related neurodegeneration and making it a good model to find the molecular mechanism of pathological aging in the brain.

One of the hallmarks of aging that has also been implicated in neurodegeneration is the impairment of mitochondrial activity. Mitochondria are vital cell organelles with many functions, including adenosine triphosphate (ATP) synthesis, calcium homeostasis handling, and lipid metabolism. ATP production occurs on the inner mitochondrial membrane, which incorporates five specific protein complexes (complexes I–V), forming the electron transport chain. The mammalian mitochondrial protein biosynthesis system involves genes from both nuclear and mitochondrial genomes. While mtDNA encodes only a small fraction of mitochondrial genes compared to nuclear DNA (1%), they are all necessary for synthesizing the respiratory complex proteins. To generate energy, electrons are transported through complexes I-IV moving across an electrochemical gradient to the ultimate acceptor, oxygen. This process is called oxidative phosphorylation (OXPHOS). As part of ATP production, various metabolites are formed in the mitochondria, such as *Acetyl-CoA*, *Citric Acid*, *Oxoglutaric acid*, *Succinic Acid*, *Malate*, and *Fumarate*. These metabolites control mitochondrial bioenergetics, and their altered levels might result in the deregulation of several aging-related pathways (e.g., mTOR, AMPK), implicating mitochondrial bioenergetic defects in aging [25–27]. During oxidative phosphorylation, the mitochondria also generate reactive oxygen species (ROS) molecules as a byproduct of ATP synthesis [28]. These molecules induce damage, which accumulates throughout the organismal lifespan and becomes harmful at high concentrations, inducing oxidative stress, DNA damage, and lipid peroxidation [29]. Since mtDNA is located near the ROS production sites, it might be more sensitive to oxidative damage and prone to possible mutations. The brain is particularly vulnerable to age-related mitochondrial damage because of its high energy demand [30]. Age-related accumulation of mitochondrial abnormalities disrupts synaptic transmission and neuronal metabolism, leading to neurodegeneration [31, 32]. However, despite the clear role of mitochondrial dysfunction as a key marker of aging and neurodegenerative diseases, the exact mechanisms initiating this dysfunction are still poorly understood.

In this study, we identify an essential function of SIRT6 in regulating the mitochondrial processes in the brain, including oxidative phosphorylation and aerobic respiration. By using transcriptomics and metabolomics profiles of control and brain-specific SIRT6-deficient mice, we demonstrate a reduction in the expression of OXPHOS-related genes, as well as the abundance of tricarboxylic acid cycle (TCA) metabolites. We functionally validate these findings by measuring the mitochondrial membrane potential and mitochondrial content changes. To establish the regulatory mechanisms by which SIRT6 affects mitochondria, we focus on YY1, SIRT3, and SIRT4 proteins. YY1 transcription factor is implicated in the regulation of mitochondria-related genes in skeletal muscle [33] and was shown to share many cellular functions with SIRT6, including those related to neurodegeneration [24]. In turn, SIRT3 is reported to be a key deacetylase in the mitochondria, targeting OXPHOS, TCA cycle, and mitochondrial dynamics [34]. Because of these abilities, SIRT3 can contribute to the protection against oxidative stress, preventing neuronal cell death [35]. Finally, we link transcriptional changes of mitochondria-related genes with normal and pathological brain aging.

## Results

### Lack of SIRT6 alters gene expression levels in the mouse brain

**Fig. 1:**
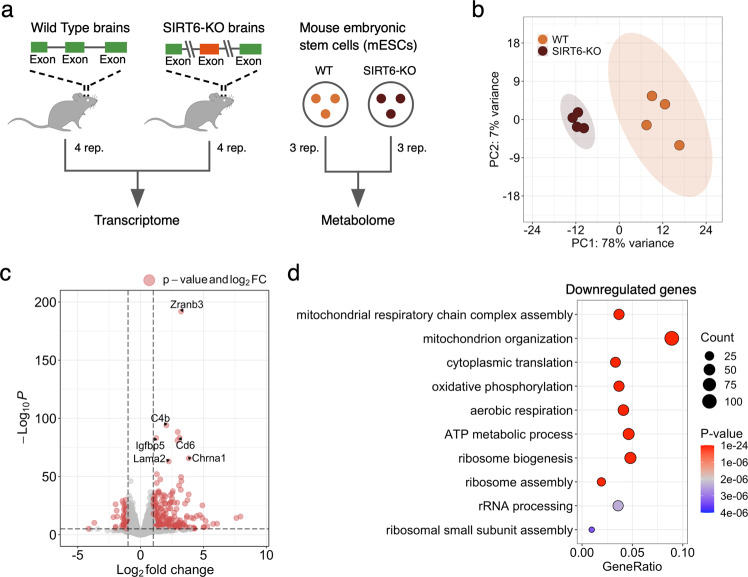
SIRT6 regulates gene expression levels. **a** Schematic illustration of the experimental design used in this study. Transcriptome profiles were collected from Wild Type (WT) and SIRT6-knockout (brSIRT6-KO) mouse brain samples. WT and brSIRT6-KO SH-SY5Y metabolomic profiles were complemented with metabolomics data on mouse embryonic stem cells. **b** Principal Component Analysis (PCA) plot showing separation between WT (orange) and brSIRT6-KO (brown) samples. Orange and brown ovals represent confidence ellipses of WT and brSIRT6-KO groups. **c** The volcano plot showing up- and downregulated differentially expressed genes in brSIRT6-KO mice compared to WT mice. Red dots indicate significantly changed genes, and gray dots represent insignificant genes. **d** GO analysis showing the top 10 enriched biological processes for downregulated genes. Each circle corresponds to the enriched GO term and varies in size according to the number of significant genes belonging to this term. The gene ratio represents the number of DE genes belonging to the enrichment categories divided by the total number of genes per category.

Brains missing SIRT6 functionality might present changes at multiple levels of molecular organization, from gene expression to metabolism. To explore these changes, we performed transcriptome profiling (RNA-seq) in brains derived from Wild Type (WT, *n* = 4) and brain-specific SIRT6-knockout (brSIRT6-KO, *n* = 4) mice (Supplementary Tables 1, 2). In addition, we applied LC–MS techniques to quantify the abundance of 34 metabolites in WT (*n* = 3) and SIRT6-KO (*n* = 3) replicates derived from the SH-SY5Y cell line and complemented them with mouse Embryonic Stem Cell (mESC) metabolomics data. Then, we conducted a multilayer bioinformatics analysis of WT and SIRT6-KO transcriptomic and metabolomic profiles (Fig. 1a).

Principal Component Analysis (PCA) of transcriptomic profiles revealed significant changes in gene expression levels between brSIRT6-KO and WT samples with a clear separation by the first principal component explaining 78% of the total variance (Fig. 1b). At the same time, transcriptomic profiles exhibited a high level of intra-group similarity, showing the Pearson’s R > 0.9986 for the WT group and Pearson’s R > 0.9992 for brSIRT6-KO replicates. In contrast, the inter-group Pearson’s R did not exceed 0.9978 (Supplementary Fig. S1a). Differential expression analysis between WT and brSIRT6-KO resulted in 2870 differentially expressed (DE) genes, ~85% of which were annotated as protein-coding sequences (Supplementary Table 3, Supplementary Fig. S1b). Consistent with the expected impaired deacetylase activity of SIRT6 upon knockout, 1481 DE genes exhibited elevated expression levels in brSIRT6-KO samples, while 1389 genes were downregulated (Fig. 1c). The list of top 10 significant features was represented exclusively by upregulated genes, including *Zranb3* (FDR *p* value = 1.32 × 10^−192^), *C4b* (FDR *p* value = 8.8 × 10^−95^), *Cd6* (FDR *p* value = 4.18 × 10^−84^), as well as *Chrna1* (FDR *p* value = 2.47 × 10^−66^) and *Lama2* (FDR *p* value = 9.95 × 10^−64^) (Supplementary Fig. S1c), which were previously found among the most significant signatures of SIRT6 deficiency in the brains of the full-body KO [24]. These results collectively indicate that SIRT6 deficiency has a major effect on transcriptional regulation in the mouse brain.

We further examined the functional roles of significant DE genes. GO enrichment analysis on upregulated genes revealed enriched terms associated particularly with ‘*external encapsulating structure organization*’ (FDR *p* value = 3.7 × 10^−08^), ‘*axon guidance*’, and ‘*neuron projection guidance*’ (FDR *p* value = 5.42 × 10^−08^ for both terms) (Supplementary Fig. 1d). Conversely, the list of downregulated features in WT compared to brSIRT6-KO was significantly enriched in genes functionally related to mitochondrial processes (Fig. 1d, Supplementary Table 4): ‘*mitochondrial respiratory chain complex assembly*’ (FDR *p* value = 1.21 × 10^−20^), ‘*mitochondrion organization*’ (FDR *p* value = 9.05 × 10^−19^), ‘*cytoplasmic translation*’ (FDR *p* value = 5.06 × 10^−17^), and ‘*oxidative phosphorylation*’ (FDR *p* value = 6.60 × 10^−17^). Overall, our findings show that SIRT6 deficiency provokes significant gene expression changes in the mouse brain and induces transcriptional dysregulation of mitochondria-related genes.

### SIRT6 regulates mitochondrial metabolism

**Fig. 2:**
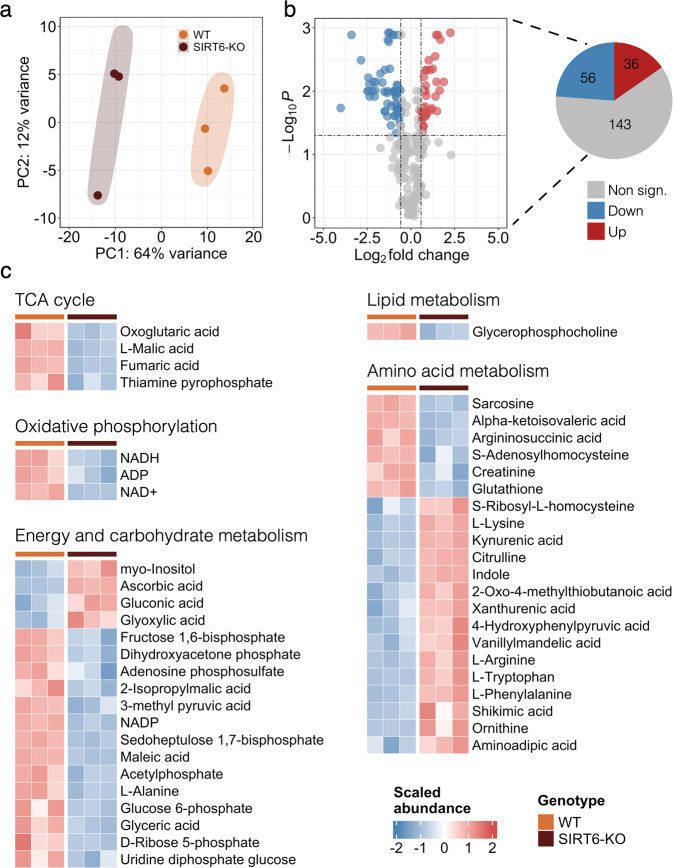
SIRT6 deficiency triggers an abundance of energy metabolites. **a** PCA plot showing separation between the WT (orange circles) and SIRT6-KO (brown circles) groups based on the mESC metabolomic profiles. Orange and brown ovals represent confidence ellipses of WT and SIRT6-KO groups. **b** The volcano plot illustrating differentially abundant metabolites detected between WT and SIRT6-KO mESC samples. Up- and downregulated metabolites are represented by red and blue circles, respectively. The pie plot (on the right) demonstrates the number of upregulated (red), downregulated (blue), and insignificant (gray) metabolites in the analysis. **c** The abundance heatmap of 68 out of 92 significant metabolites classified according to the metabolic pathways they are involved in.

Based on the significant association of DE genes with essential mitochondrial processes, we wondered whether SIRT6 silencing might induce alterations in mitochondrial metabolite levels. To study the role of SIRT6 in mitochondrial metabolism, we examined the differential metabolite abundance patterns in SIRT6-KO untargeted LC–MS profiles compared to WT in the mouse embryonic stem cells data (Supplementary Table 5). Similar to RNA-seq results, we observed a global difference between the abundance levels of WT and SIRT6-KO metabolite profiles, underlined by their clear separation by PC1 in the PCA plot (Fig. 2a). Differential abundance (DA) analysis revealed that 92 out of 235 metabolomic features (~39%, FDR < 0.05 and |log_2_ (Fold Change)| > 0.58) changed significantly between experimental conditions (Fig. 2b), including *Ascorbic acid* (upregulated), *Maleic acid* (downregulated), and *NAD*^*+*^ (downregulated) as the most significant metabolites (Supplementary Fig. S2a, b). Consistent with the transcriptome analysis, we found a number of DA features related to mitochondrial energy system pathways. Several metabolites associated with catabolic processes were more abundant in the SIRT6-WT group compared with SIRT6-KO: four metabolites (*Malic acid*, *Fumaric acid*, *Oxoglutaric acid*, *Thiamine Pyrophosphate*) associated with TCA cycle and three metabolites (*NAD*^*+*^, *NADH*, *ADP*) associated with OXPHOS (Fig. 2c). The same tendency was observed for other DA metabolites related to the energy and carbohydrate metabolic pathways, of which only four metabolites were upregulated, while the rest fourteen were decreased in SIRT6-KO. In addition to these results, we found abundant alterations of metabolomic features that constitute the *Lipid* and *Amino Acid metabolism* pathways. Thus, our results show that SIRT6 silencing alters cellular and mitochondrial metabolism.

### SIRT6 deficiency leads to impaired oxidative phosphorylation

**Fig. 3:**
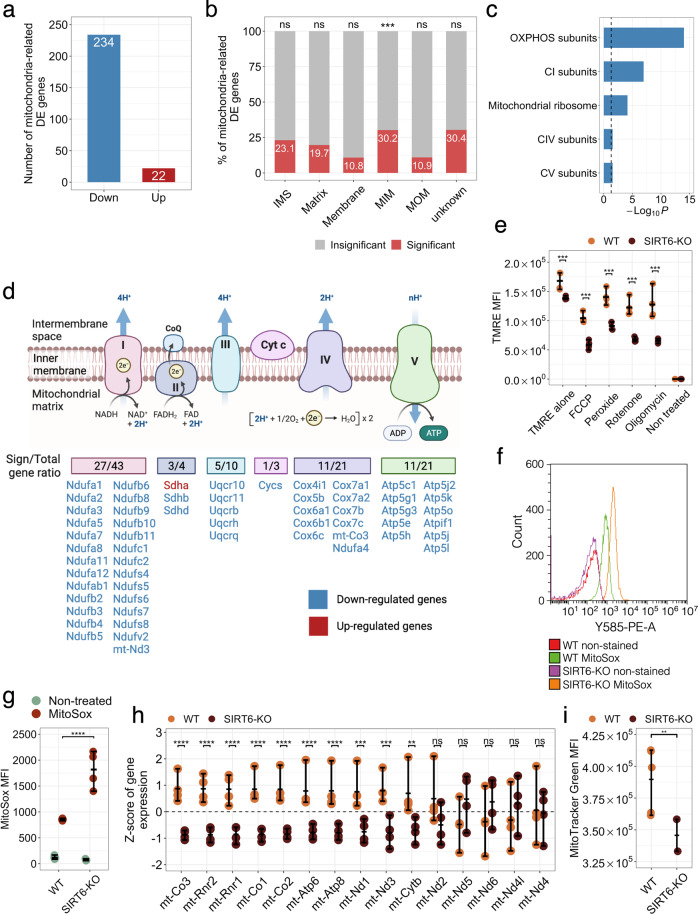
OXPHOS impairment in SIRT6-KO. **a** Number of significant DE genes associated with mitochondrial functions in WT compared to SIRT6-KO. Red and blue bars show the number of up- and downregulated genes. **b** Percentage of significant (red bars) and insignificant (gray bars) genes across mitochondrial compartments. ‘IMS’ denotes intermembrane space, ‘MIM’ denotes mitochondrial inner membrane, and ‘MOM’ corresponds to the mitochondrial outer membrane. Black asterisks indicate the statistical significance of the enrichment (*p* value = 5.5 × 10^−04^, hypergeometric test). **c** Overrepresented mitochondrial pathways for WT compared to SIRT6-KO. The statistical significance threshold of 0.05 (hypergeometric test) is shown by a black dashed line. **d** Schematic illustration of the ratio between the number of significant DE genes associated with Cytochrome C oxidase and I–V complexes of the electron transport chain and the total number of genes per complex. Down- and upregulated genes are marked by blue and red colors, respectively. **e** Mitochondrial membrane potential measured in SH-SY5Y WT and SIRT6-KO cells under treatment with FCCP (10 µM), Hydrogen peroxide (800 nM), Rotenone (200 nM), Oligomycin (20 µM), and without treatment. Asterisks indicate the level of statistical significance (*p* < 0.05, t-test). **f** Mitochondrial ROS detection with MitoSox assay. The histogram shows fluorescence emission distributions measured in WT and SIRT6-KO cells that were non-stained and MitoSox-treated. Distribution of mean fluorescence intensity (MFI) values measured in WT and SIRT6-KO cells that were non-treated (green circles) and MitoSox-treated (red circles). Error bars are mean ± SD, ****p < 0.0001 (*n* = 3–5). **g**,**h** Z-score transformed expression levels of mtDNA genes detected in our RNA-seq experiment. Orange and brown circles represent WT and SIRT6-KO samples, respectively. **i** Difference in mitochondrial content between WT and SIRT6-KO SH-SY5Y cells. Asterisks indicate the level of statistical significance (*p* < 0.05, t-test).

Furthermore, we focused on the significant DE mitochondrial genes that affected mitochondrial pathways. SIRT6 deficiency resulted in 256 significant DE mitochondria-related genes out of 1140 genes with confirmed mitochondrial localization according to the MitoCarta database [36] (Fig. 3a). Importantly, downregulated genes constituted the majority (>91%) of all DE mitochondria-related genes. Of note, protein levels of one of the most significantly downregulated genes (*Cycs*, FDR *p* value = 3.00 × 10^−19^) were consistently decreased in both male and female SIRT6-KO brains (Supplementary Fig. S3a, b). On the contrary, both expression and protein levels of *Vdac1* changed insignificantly between female WT and SIRT6-KO samples but showed a reduction in protein levels in male SIRT6-deficient brains (Supplementary Fig. S3a, b). Interestingly, we found that mitochondrial genes were overrepresented in their localization at the Mitochondrial Inner Membrane (MIM) compartment (Fig. 3b, Supplementary Fig. S3c). Given that MIM serves as a springboard for ATP synthesis, we hypothesize that significant mitochondria-related genes should be mostly associated with electron transport chain complexes. To explore biological functions related to DE mitochondrial genes, we performed GO enrichment analysis using information about mitochondrial pathways obtained from MitoCarta as a specific background. The top enriched categories included terms associated with mitochondrial respiratory chain complexes and mitochondrial ribosomes (Fig. 3c). Mitochondrial Complex I turned out to be the most affected by SIRT6 depletion (FDR *p* value = 1.09 × 10^−07^), with 27 downregulated out of 43 genes encoding this Complex. Of note, 57 out of 99 genes encoding the electron transport chain subunits were differentially expressed in our analysis. But only *Succinate dehydrogenase complex flavoprotein subunit A* gene (*Sdha*) demonstrated an elevated level of expression (Fig. 3d). Also, we confirmed that these changes also occur in brain RNA-seq samples of two human donors from Allen Brain Atlas [37], where the correlation between the expression of SIRT6 and the expression of OXPHOS-related genes is significantly stronger (*p* value = 0.000636 and *p* value = 0.000002, respectively) compared to other mitochondria-related genes (Supplementary Fig. S3d).

We hypothesized that a global reduction in the expression of OXPHOS genes and electron transport chain (ETC) complex activity in SIRT6-KO models might be accompanied by the corresponding decline in mitochondrial membrane potential ΔѰ. In order to check this hypothesis, we first measured ΔѰ in WT and SIRT6-deficient SH-SY5Y cells stained with TMRE dye. Indeed, SIRT6-KO mitochondria showed a significant 1.21-fold decrease in ΔѰ compared to WT cells (FDR *p* value = 0.0006, Tukey’s multiple comparisons test, Fig. 3e, Supplementary Table 6). Then, we tested ΔѰ in the same WT and SIRT6-KO cells but treated with an uncoupler of oxidative phosphorylation FCCP. Interestingly, supplementation of FCCP enhanced the reduction effect of ΔѰ upon SIRT6 deficiency, resulting in 1.78-fold decrease of ΔѰ in SIRT6-KO cells (FDR *p* value = 0.0001, Tukey’s multiple comparisons test). Similar significant changes were observed when inhibitors of individual complexes of the ETC were added to the cells. SIRT6-KO cells with inactivated Cytochrome C complex by hydrogen peroxide showed 1.54-fold reduction in ΔѰ (FDR *p* value = 0.0001, Tukey’s multiple comparisons test), while mitochondria with inactivated Complex I (rotenone treatment) and ATP synthase (oligomycin treatment) showed the highest level of ΔѰ reduction in SIRT6-KO, in 1.81 and 1.93 times, respectively (FDR *p* value = 0.0001 in both cases, Tukey’s multiple comparisons test), suggesting higher dependence of SIRT6 for these complexes. Then, we speculated that an elevated ROS production could also accompany observed transcriptional changes of OXPHOS-related genes and ΔѰ reduction upon SIRT6 knockout. Indeed, using MitoSox staining, we detected significantly increased levels of ROS in SIRT6-KO cells compared to WT (Fig. 3f-g). All these results collectively indicate that the mitochondrial oxidative phosphorylation process is markedly impaired in SIRT6-deficient cells.

### Lack of SIRT6 results in a reduction of mtDNA gene expression and mitochondrial content

Mitochondrial activity is regulated by both nuclear and mitochondrial DNA encoded genes. Since all mitochondrial-encoded genes are involved in oxidative phosphorylation, we studied the expression changes of these genes in SIRT6-KO brains. In particular, we extracted the expression of fifteen mtDNA genes detected in our RNA-seq data and the direction of their expression changes in WT and SIRT6-KO samples. Four out of these fifteen mtDNA genes were downregulated in SIRT6-KO mice, including statistically significant genes *mt-Co3* (FDR *p* value = 3.8 × 10^−18^), *mt-Rnr2* (FDR *p* value = 1.1 × 10^−14^), *mt-Rnr1* (FDR *p* value = 1.0 × 10^−11^), *mt-Nd3* (FDR *p* value = 5.2 × 10^−04^) (Fig. 3h). In addition, six other mtDNA-encoded genes (*mt-Co1*, *mt-Co2*, *mt-Atp6*, *mt-Atp8*, *mt-Nd1*, *mt-Cytb*) showed a statistically significant reduction in expression (FDR < 0.05), but did not meet log_2_ (Fold Change) criterion for significance. Since altered mtDNA gene expression levels might indicate co-directional changes in mitochondrial content, we also measured mitochondrial mass in WT and SIRT6-KO SH-SY5Y cells using the MitoTracker Green assay. Consistent with transcriptional downregulation patterns of mtDNA genes, mitochondrial mass was significantly lower in SIRT6-deficient cells (~21.8% decrease, T-test *p* value = 0.0087) than in WT cells (Fig. 3i, Supplementary Table 6), which in turn can be a marker of impaired mitochondrial biogenesis or increased degradation.

### SIRT6-SIRT3,4 and SIRT6-YY1 axes promote OXPHOS in the brain

**Fig. 4:**
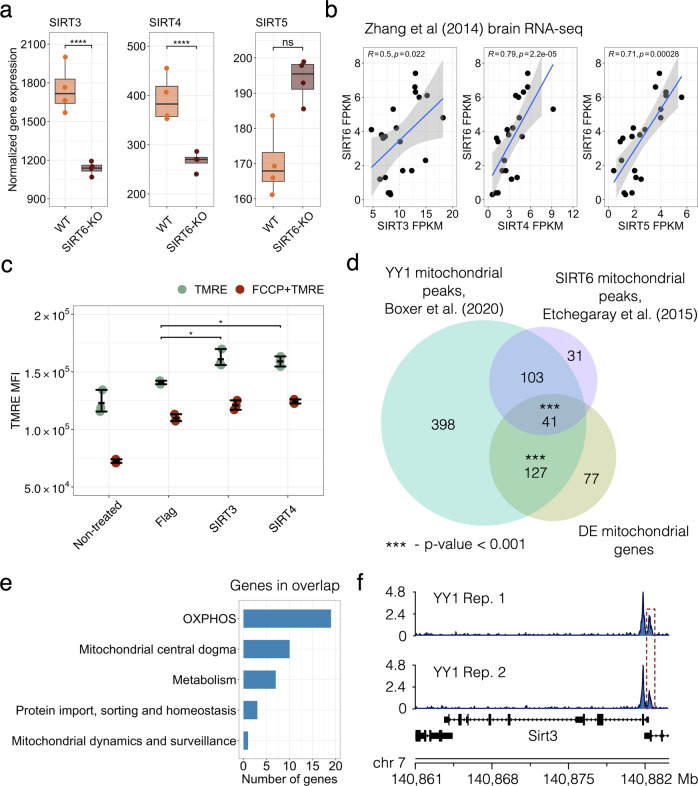
SIRT6-SIRT4 and SIRT6-YY1 axes in mitochondrial regulation. **a** SIRT3-5 expression levels in transcriptomic profiles of WT and SIRT6-KO mice. Asterisks indicate the statistical significance (FDR *p* value < 0.05) derived from DESeq2 differential expression analysis. **b** Spearman’s correlation coefficients of SIRT6 expression profile with expression profiles of mitochondrial sirtuins (SIRT3-5) in the brain RNA-seq data from Zhang et al. [39]. **c** Mitochondrial membrane potential measured in SH-SY5Y SIRT6-KO cells when SIRT3 or SIRT4 were exogenously overexpressed. SIRT6-KO SH-SY5Y cells were transfected with Flag-CMV, SIRT3-Flag-CMV, and SIRT4-Flag-CMV plasmids. After 48 h, cells were collected and stained with TMRE and Life/dead viability dye and the intensity of fluorescence was measured by CytoFLEX. **d** Venn diagram showing overlaps between significant mitochondria-related genes from the RNA-seq analysis (orange), YY1 mitochondrial targets (green) and SIRT6 mitochondrial targets (purple). Statistical significance of the overlaps was calculated using the permutation test. **e** Bar plot showing biological functions along with the number of the mitochondria-related genes overlapped between all datasets presented in (**d**). **f** YY1 peaks at SIRT3 promoter in two analyzed ChIP-seq replicates.

Next, we elucidated the mechanism behind the SIRT6-dependent regulation of mitochondrial activity and the oxidative phosphorylation process. First, we explored SIRT3, SIRT4, and SIRT5 genes from the sirtuin family, which encode proteins localized in mitochondria and coordinately impact mitochondrial pathways related to redox homeostasis and cellular metabolism [38]. To determine whether SIRT6 may transcriptionally regulate these genes, we examined their expression patterns upon SIRT6 knockout (Fig. 4a). SIRT3 and SIRT4 were significantly reduced in SIRT6-KO brains (FDR *p* value = 3.60 × 10^−12^ and 3.33 × 10^−06^, respectively). At the same time, the lack of SIRT6 did not substantially affect SIRT5 expression. We further confirmed the positive association between SIRT6 and SIRT3-4 by analyzing publicly available gene expression data in the mouse brain from Zhang et al. [39] (Fig. 4b). SIRT6 expression levels positively correlated with the corresponding expression levels of all mitochondrial sirtuins (Pearson’s R = 0.5, 0.79, 0.71 for correlations with SIRT3, SIRT4, SIRT5, respectively). Then, we focused on SIRT3 and SIRT4 genes, which most significantly changed among mitochondrial sirtuins. To experimentally validate their role in OXPHOS regulation, we assessed the changes in mitochondrial membrane potential ΔѰ in SIRT6-KO SH-SY5Y cells with overexpressed SIRT3 and SIRT4. We found that increased expression of SIRT3 or SIRT4 significantly rescued ΔѰ in SIRT6-deficient cells compared to that in WT cells suggesting their importance for the regulation of oxidative phosphorylation when SIRT6 is absent (Fig. 4c).

Given that SIRT6 is involved in the regulation of distinct cellular functions, we next wondered whether the transcription regulation of mitochondrial OXPHOS by SIRT6 was specified by a co-regulating transcription factor YY1. We have previously shown that SIRT6 and YY1 functionally interact by forming a complex that regulates several shared target genes [24]. To examine whether they might regulate mitochondrial processes in a coordinated manner, we analyzed two publicly available YY1 ChIP-seq datasets in cortical neurons (GSE128182 GEO accession). We searched for YY1 peaks corresponding to the promoters of mitochondria-related genes. In addition, we compared these peaks with both mitochondria-related DE genes from our RNA-seq analysis as well as with SIRT6 targets involved in mitochondrial regulation derived from public mESC ChIP-seq profiles (GSE65836). As a result, we detected 669 YY1 peaks associated with promoters of mitochondrial genes, including 168 peaks that were localized within 1 kb from the promoters of mitochondrial DE genes and 144 peaks colocalized with SIRT6 binding sites in mESC (Fig. 4d). We also identified only 11 SIRT6 binding sites in the absence of YY1 peaks at mitochondria-related gene promoters, also suggesting a smaller indirect mechanism of mitochondrial regulation by SIRT6.

Interestingly, both YY1 and SIRT6 peaks were overrepresented at the promoters of genes localized in the mitochondrial protein-containing complex (FDR *p* value = 1.01 × 10^−38^ and FDR *p* value = 3.96 × 10^−12^, respectively) and the mitochondrial inner membrane (FDR *p* value = 1.82 × 10^–27^ and FDR *p* value = 2.66 × 10^−08^, respectively), while YY1 target genes were also enriched for mitochondrial matrix (FDR *p* value = 3.41 × 10^–^^37^) and ATPase complex (FDR *p* value = 4.34 × 10^−22^) (Supplementary Fig. S4a, b). Our analysis revealed that the expression of more than 66% of the detected mitochondria-related genes could be regulated by either YY1 or by YY1 and SIRT6 together. Both YY1 and SIRT6 peaks were found within promoters of 41 mitochondria-related DE genes that were also overrepresented (permutation test *p* value = 5.1 × 10^−04^) in this overlap compared to non-significant mitochondria-related genes. These genes are also related to OXPHOS, mitochondrial metabolism, and protein import regulation (Fig. 4e, Supplementary Fig. S4c). Besides its coordinated regulatory activity with SIRT6, YY1 can also independently bind to the promoters of mitochondria-related DE genes. In our analysis, it was enriched (permutation test *p* value = 1.0 × 10^−03^) at the promoters of such 127 DE genes, including SIRT3 (Fig. 4f), which importance for OXPHOS was shown above. Hence, our analysis suggests that SIRT6 acts as a regulator of mitochondrial functions via the SIRT6-YY1-SIRT3,4 axis.

### Neuropathological role of SIRT6 through the prism of mitochondrial deregulation

**Fig. 5:**
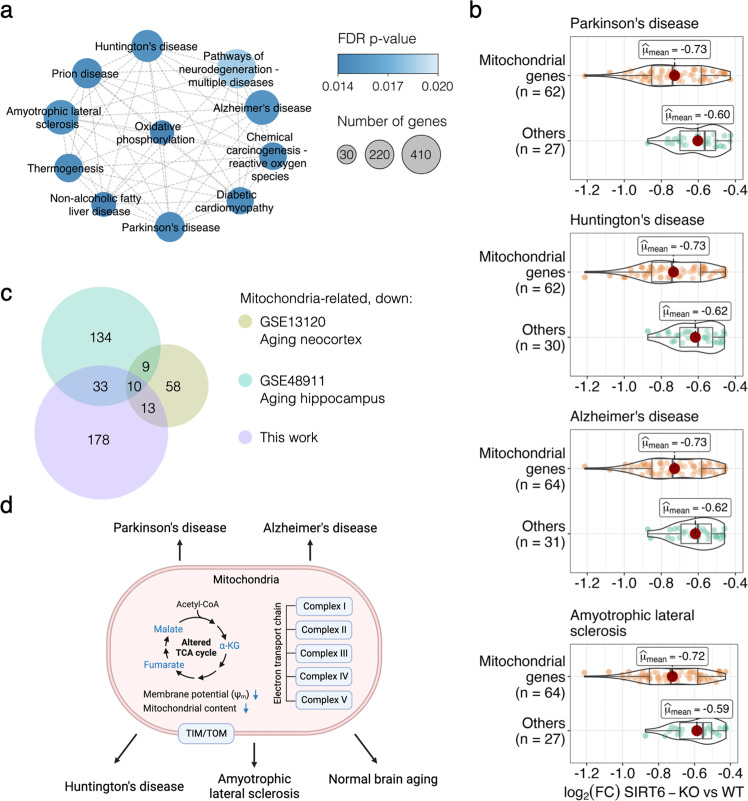
SIRT6 silencing triggers neurodegenerative disease pathways and normal brain aging. **a** Cluster of enriched KEGG pathways obtained using GSEA. Each circle represents an enriched pathway and is colored according to the FDR *p* value. **b** Violin plots representing the log_2_ (Fold Change) expression for genes with the largest contribution to the GSEA enrichment result per neurodegenerative pathway. **c** Euler diagram showing ten common downregulated genes between publicly available aging brain gene expression datasets (GSE13120 and GSE48911 accessions in the GEO database) and significantly downregulated mitochondrial genes from this study. **d** Proposed model of the mitochondrial dysfunction caused by SIRT6 silencing and its involvement in neurodegenerative diseases and normal aging. The figure was generated using the BioRender website.

Sirtuin 6 has been reported to be important in the protection against age-related and neurodegenerative diseases in the brain [18, 23, 40, 41]. Since, in our analysis, we observed a global reduction in the transcriptional level of mitochondrial genes, we explored whether these changes can be linked to pathways of age-associated diseases occurring in the brain. Therefore, we performed the Gene Set Enrichment Analysis (GSEA) based on all genes in our RNA-seq dataset. This analysis revealed 71 significantly affected KEGG pathways (Supplementary Table 7, Supplementary Fig. S5), including ‘Parkinson’s disease’ (FDR *p* value = 0.015), ‘Huntington’s disease’ (FDR *p* value = 0.0168), ‘Alzheimer’s disease’ (FDR *p* value = 0.0169), and ‘Amyotrophic lateral sclerosis’ (FDR *p* value = 0.0168) pathways (Supplementary Fig. S5). Interestingly, these neurodegenerative disease pathways formed one distinct cluster with ‘Oxidative phosphorylation’ pathway in the enrichment network, showing a large number of overlapping genes between them (Fig. 5a). To address whether the expression changes of mitochondria-related transcripts directly caused the enrichment of these pathways, we retrieved core enrichment genes from the pathways of interest. More than 67% of core enrichment genes in each selected pathway were associated with mitochondrial functions. The highest percentage was detected for Alzheimer’s disease (Fig. 5b). Moreover, the mitochondria-related core enrichment genes exhibited lower mean log_2_ (Fold Change) values compared to non-mitochondrial genes in each of the selected neurodegenerative disease pathways.

Since mitochondrial dysfunction is one of the most stable and crucial hallmarks of normal aging [25, 26, 42], we then compared our downregulated mitochondria-related genes with genes that were previously reported to be signatures of mouse brain aging [43, 44]. As a result, we captured ten downregulated mitochondrial genes that also showed a reduction in their expression levels in both neocortex and hippocampus aging data (Fig. 5c). Notably, this list of commonly downregulated genes included genes related to OXPHOS complexes (*Sdhd*, *Ndufa7*, *Uqcrq*) and mitochondrial protein import machinery (*Timm10b*). Another interesting overlapping gene was *Uracil DNA Glycosylase* (*Ung)*, which has an important role in mitochondrial base excision repair (BER) initiation. Given the limited DNA repair mechanisms in mitochondria, one can expect that the decrease in *Ung* activity might provoke an accumulation of mutations in mtDNA. Taken together, the findings reported above suggest that the brain lacking SIRT6 expression is characterized by mitochondrial dysfunction (OXPHOS impairment, TCA dysregulation, reduced mitochondrial content and membrane potential) that causes a neurodegenerative-like phenotype and contributes to pathological aging of the brain (Fig. 5d).

## Discussion

In this study, we performed RNA-seq and LC–MS experiments to trace molecular organization changes at transcriptome and metabolome levels of SIRT6-knockout systems. We show that SIRT6 deficiency leads to a dramatic downregulation of mitochondrial genes and changes in mitochondria-related metabolite composition, suggesting that SIRT6 critically regulates mitochondrial activity in the brain.

Our analysis of gene expression levels in the SIRT6-deficient mouse brain revealed dramatic transformations upon SIRT6 knockout: almost three thousand genes changed their expression significantly, with a strong enrichment of mitochondrial functions among downregulated ones, allowing us to conclude that SIRT6-knockout induces transcriptional dysregulation of mitochondrial genes. This result bridges the missing gap between studies demonstrating mitochondrial dysfunction in normal and pathological aging [1, 45] and studies proclaiming the critical role of SIRT6 in the protection against aging-associated diseases [18, 19].

Though mitochondrial dysfunction is a known marker of aging and neurodegenerative diseases, the exact mechanism behind it remains unknown. Our study suggests that the decay of SIRT6 levels during aging [18] and in Alzheimer’s disease [18, 23, 46] could be a key mechanism causing the deterioration of mitochondrial functions. The changes induced by the SIRT6 knockout that we observe at the metabolite level support this claim: metabolites related to mitochondrial energy system pathways (in particular, OXPHOS and TCA cycle) are significantly overrepresented among differentially abundant metabolites. In line with the discussed mitochondrial dysfunction in aging, all these metabolites are downregulated in the SIRT6-KO samples. Importantly, the dramatic decline of one of them, NAD^+^, was also associated with pro-senescence mechanisms in various species [47, 48], as well as with limited neuroprotective activity of sirtuins [49].

Accordingly, the vast majority of differentially expressed mitochondria-related genes were downregulated in our gene expression analysis. As they were strongly enriched with mitochondrial respiratory chain complexes, we measured the mitochondrial membrane potential and mitochondrial content in SIRT6-KO cells because reduced gene expression might indicate the loss of mitochondria. Both measured characteristics were significantly decreased, validating the suggested impairment of mitochondrial oxidative phosphorylation and mitochondrial biogenesis in SIRT6-deficient brains. Interestingly, the average decrease of mtDNA gene expression (~19.7%) in SIRT6-KO was in good agreement with the corresponding reduction of mitochondrial content (21.8%), suggesting impaired mitochondrial biogenesis as a primary cause of the observed transcriptional dysregulation in mitochondria upon SIRT6 knockout.

Concordantly, the impaired membrane potential upon SIRT6-KO can be partially rescued by restoring SIRT3 and SIRT4 levels, which were significantly downregulated in SIRT6-deficient brains. Both of them are localized in mitochondria and impact mitochondrial pathways related to redox homeostasis and cellular metabolism [38] and have important roles in mitochondria metabolism ROS balance and lifespan [50–52]. The analysis of our and publicly available gene expression data [39] confirms that SIRT6 transcriptionally regulates SIRT3 and SIRT4. Our analysis further indicates that SIRT6 regulates mitochondrial gene expression through the transcription factor YY1. We have previously shown that SIRT6 and YY1 form a complex that regulates many shared target genes [24]. Our analysis of YY1 ChIP-seq data [53] suggests that SIRT6 and YY1 regulate mitochondrial processes coordinately.

Taken together, our results establish that SIRT6 knockout induces global molecular transformations in the brain: almost three thousand genes change their expression significantly, as well as nearly half of all studied metabolites. Part of these differences are distinctly attributed to mitochondrial dysfunction, particularly in mitochondrial respiratory chain complexes, as confirmed by measurements of the mitochondrial membrane potential and mitochondrial content. Our data suggest that SIRT6 regulates mitochondrial function through YY1 and SIRT4. Lastly, we reveal that the changes driven by SIRT6 loss also occur in neurodegenerative diseases and aging brains, suggesting that age-dependent SIRT6 decay plays a causal role in neurodegenerative diseases. Preventing the reduction of SIRT6 expression or augmenting SIRT6 activity might thus provide a therapeutic opportunity to reinstate mitochondrial homeostasis in the brain and prevent neurodegenerative diseases.

## Materials and methods

### Generation of brain-specific SIRT6-KO mice and cells

SIRT6-KO mice and SH-SY5Y SIRT6-KO cells were generated using the protocol described in Sebastián et al. [54] and Kaluski et al. [18].

### RNA preparation and quality control

RNA was extracted from mice’s left brain hemispheres, using the NucleoSpin RNA Plus kit (MACHEREY-NAGEL GmbH & Co. K.G., catalog number 740984.50), according to the manufacturer’s manual. The purified RNA was then cleaned from any possible residual genomic DNA contamination using the RNeasy MinElute Cleanup Kit (QIAGEN, catalog number 74204), according to the manufacturer’s manual. Using TapeStation, RNA Integrity Number (RIN) was then assessed and only samples with RIN > 8.7 were in use.

### Full-length poly-A RNA sequencing

Library preparation was conducted by The Crown Genomics Institute of the Nancy and Stephen Grand Israel National Center for Personalized Medicine, Weizmann Institute of Science, Israel (G-INCPM). Briefly, the library kit used was the in-house INCPM mRNA-seq kit (G-INCPM, Weizmann Institute of Science), for full-length RNA-seq with polyA-based capturing. Sequencing was done using 4 lanes of NextSeq 500 High Output v2.5 Kit (75 cycles) (Illumina Inc., catalog number 20024906).

### RNA-seq data processing

Raw reads from eight *M. musculus* RNA samples were filtered and trimmed using *fastp* [55] and then processed via version 3.0 of the *nf-core/rnaseq* pipeline [56]. In brief, trimmed reads were filtered with the *Trim Galore!* tool and mapped to the mouse GRCm39 reference genome with *STAR* [57]. Then, gene expression was quantified using the *Salmon* tool [58]. The full guidelines for the pipeline are available at https://nf-co.re/rnaseq. Gene annotation was performed using the *AnnotationDbi* R package [59] with downloaded *EnsDb.Mmusculus.v79* annotation database [60] generated from Ensembl.

### Differential expression analysis

Differential expression (DE) analysis was performed via the DESeq2 [61] package in the R programming language. First, we removed low-expressed genes for which the minimum expression level within any group of samples was <3. Then, raw gene counts were normalized using DESeq2’s median of ratios method, and quality control procedures were performed. The following design formula was used to evaluate expression differences between groups of samples: *design* = *~genotype*. After fitting the Negative Binomial model for each gene, we performed pairwise comparisons between groups using the Wald test. Genes were considered to be differentially expressed if *FDR p value* < 0.05 and |*log2 (Fold Change)*| > 1.5.

### Functional analysis of genes

We used *clusterProfiler* R package [62] to perform Gene Ontology (GO) enrichment analysis on both sets of down- and upregulated genes. Redundant GO categories were removed using the ‘*simplify*’ function from *clusterProfiler* package with default settings. Gene Set Enrichment Analysis (GSEA) was performed with *gseaKEGG* function from *clusterProfiler* and pairwise similarity of significant KEGG pathways was calculated with *pairwise_termsim* function from *enrichplot* package [63] using the Jaccard similarity measure. Then, the pathway similarity network was constructed and visualized in *Cytoscape* [64]. Next, core enrichment genes for four pathways related to neurodegenerative diseases (Parkinson’s disease, Huntington’s disease, Alzheimer’s disease, Amyotrophic lateral sclerosis) were retrieved and classified into two groups according to their relevance to mitochondria (‘mitochondrial genes’ and ‘others’ groups). Expression level distributions for these two groups were visualized using *ggstatsplot* R package [65].

### Analysis of mitochondria-related genes

A list of mouse mitochondria-related genes, as well as information regarding their sub-mitochondrial localization and related mitochondrial pathways, were obtained from the *MitoCarta* database [36] (version 3.0). A total of 149 mitochondrial pathways were used for the enrichment analysis of DE mitochondria-related genes, performed with the ‘enricher’ function from the *ClusterProfiler* package. An illustration of electron transport chain complexes with associated DE genes was performed using the *BioRender* website (https://biorender.com/).

### Extraction, measurement of metabolomics profiles and data processing

Extraction and measurement of polar metabolites from brain tissue using LC–MS were performed as described by Giavalisco et al. [66] and Lapidot‐Cohen et al. [67]. In brief, 1 ml of a homogeneous mixture of pre-cooled methanol/methyl-tert-butyl-ether/water (1:3:1) were added and vortexed. This was followed by shaking for 10 min and another 10 min of incubation in an ice-cooled ultrasonication bath. 500 μl of UPLC-grade methanol/water (1:3) was added to the homogenate, then vortexed and spun for 5 min at 4 °C. The result was a phase separation with polar and semi-polar metabolites in the lower aqueous phase. Equal volume of that phase 300 μl was taken twice: to two separate tubes, and next dried down in Speedvac and stored at −80 °C until subsequent LC–MS analysis. Prior to LC–MS analysis, samples were resuspended in 80% (v/v) methanol and 20% (v/v) water. LC–MS data were obtained using the Waters Acquity UPLC system (Waters), coupled to the Exactive mass spectrometer (Thermo Fisher Scientific). A HSS T3 C_18_ reversed-phase column (100 mm×2.1 mm×1.8μm particles; Waters) was used and the temperature was set to 40 °C. The mobile phases were 0.1% formic acid in H_2_O (Buffer A, ULC MS grade; BioSolve) and 0.1% formic acid in acetonitrile (Buffer B, ULC MS grade; BioSolve). A 5-μl sample was injected. The spectra were recorded alternating between full-scan and all-ion fragmentation-scan modes, covering a mass range from 100 to 1500 *m*/*z*. The resolution was set to 25,000, with maximum time scan 250 ms. Chromatograms from the UPLC–FT/MS runs were analyzed and processed with Compound Discoverer 3.3 (Thermo Fisher Scientific) and Xcalibur^TM^ Software (Thermo Fisher Scientific). Molecular masses, retention times, and associated peak intensities for each sample were extracted from the raw files.

### Differential abundance analysis

Metabolite differential abundance analysis was done with the *MetaboAnalyst* platform [68]. Annotated mouse ESC metabolites were normalized via the median normalization method and then were log_2_ transformed. Principal components of the data were calculated using the ‘prcomp’ function in R and then used for the visualization of the profiles. Student’s t-test was applied to define significantly changed metabolites, followed by the log_2_ (*Fold Change*) calculation. Differentially accumulated metabolites were retrieved according to *FDR* < *0.05* and |log_2_ (*Fold Change*)| > 0.58 cutoff criteria. Volcano plot visualization was done with the *EnhancedVolcano* [69] package in R. Significant features were classified by their metabolic pathway identity provided by the KEGG database [70]. *ComplexHeatmap* R package [71] was used to plot heatmaps of metabolite abundances.

### Analysis of public brain RNA-seq data

Processed and FPKM-normalized mouse brain RNA-seq profiles were downloaded from Zhang et al. [39]. Only expression levels of SIRT6 and mitochondrial sirtuins (SIRT3, SIRT4, SIRT5) were selected, followed by the Spearman correlation calculation. Analysis of the correlation of SIRT6 with mitochondria-related genes was done using brain RNA-seq data of two human donors (H0351.2001, H0351.2002) from the Allen Brain Atlas database [37]. Using the list of mitochondria-related genes retrieved from MitoCarta (version 3.0), Spearman’s correlation coefficients of SIRT6 with OXPHOS and non-OXPHOS-related genes were calculated for both donor expression profiles. Permutation test (number of permutations = 1000,000) was used to test the assumption regarding the unlikeness of distributions for OXPHOS and non-OXPHOS-related genes.

### Analysis of YY1 and SIRT6 ChIP-seq data

Processed data of two mouse YY1 ChIP-seq replicates in cortical neurons (SRX5509061 and SRX5509062 accession numbers [53]), and SIRT6 ChIP-seq replicates in mouse embryonic stem cells (SRX873340, SRX873342, SRX873343 accession numbers [54]) were downloaded from the *ChIP-Atlas* database [72]. Called peaks with q < 1 × 10^−05^ were annotated by their genome position using ‘*annotatePeak*’ function from *ChIPseeker* package [73] and only peaks localized at promoters of mitochondria-related genes in all replicates were selected. SIRT6 peaks called for both SIRT6-KO and WT replicates were subtracted from the analysis. The cellular component (CC) GO analysis of genes associated with the selected YY1 and SIRT6 peaks was performed using *ClusterProfiler*. Area-proportional Venn diagram for mitochondria-related DE genes, YY1- and SIRT6-regulated mitochondria-related genes was plotted using *venneuler* R package. The significance of overlap was calculated via a permutation test with non-significant mitochondria-related genes as a specific background. ChIP-seq profiles of the selected peaks were visualized with the *karyoploteR* package [74].

### Analysis of public aging brain datasets

Microarray gene expression data of mouse aging neocortex (five 5-month-old and five 30-month-old, GSE13120 [43]) and mouse aging hippocampus (three 10-days-old and three 20-month-old, GSE48911 [44]) were used for the analysis. Only WT replicates were selected from aging hippocampus datasets. Differential expression analysis was performed via *GEO2R* online tool [75] with default parameters.

### Quantification of mitochondrial mass

To measure the mitochondrial mass, SH-SY5Y SIRT6-WT and SIRT6-KO cells were stained with 100 nM MitoTracker Green (M7514; Molecular Probes) and viability dye (eBioscience™ Fixable Viability Dye eFluor™ 780, 65-0865-14, Invitrogen) in the dark for 30 min at 37 °C. Fluorescence intensities of Mitotracker™ Green were detected by using a flow cytometer (CytoFLEX S Flow Cytometer).

### Mitochondrial Membrane Potential Assay

To measure the mitochondrial membrane potential, SH-SY5Y SIRT6-WT and SIRT6-KO cells were treated with trypsin, washed once and 1×10^5^ cells were loaded to each well of 96-well plates. Cells were resuspended in DMEM with 10% FBS containing different concentrations of rotenone or oligomycin or hydrogen peroxide or FCCP as indicated in figure legends and incubated in the dark for 30 min in a cell culture incubator. After 30 min, cells were washed in PBS and incubated in the dark for 30 min with TMRE and viability dye at 37 °C (TMRE Mitochondrial Membrane Potential Assay Kit, 701310). Then, cells were washed and fluorescence intensities were detected by using flow cytometry (CytoFLEX S Flow Cytometer).

### Measurement of mitochondrial ROS generation

The measurement of mitochondrial ROS generation was performed by using MitoSOX™ Red staining. SH-SY5Y SIRT6-WT and SIRT6-KO cells were treated with trypsin and then centrifuged at 500 g for 5 min. Cells were washed once with HBSS and incubated with 5 μM MitoSOX™ and viability dye for 15 min at 37 °C in the dark. After treatment, cells were washed in HBSS and MitoSOX™-positive cells were detected by using flow cytometry (CytoFLEX S Flow Cytometer).

### Western blot analysis

Total protein extracts from WT (*n* = 8) and brSirt6-KO (*n* = 8) brains were prepared. Fifteen micrograms of protein were loaded onto 4–20% Tris-Glycine polyacrylamide gel (BioRad). Proteins were separated for 1 h at 120 V and then blotted to nitrocellulose membranes at 100 V for 35 min. The blots were blocked with 5% BSA in TBST (15 mM Tris-HCl, pH 7.5, 200 mM NaCl, and 0.1% Tween 20) for 1 h at room temperature. Membranes were incubated overnight with a mouse monoclonal anti-cytochrome c antibody (clone 7H8.2C12; 1:1000; PharMingen Becton Dickinson), a mouse monoclonal anti-VDAC1 antibody (Santa Cruz biotechnology, sc-390996) and anti-β-tubulin antibody (Cell Signaling, 2128). The blots were developed using the [74] chemiluminescence reagent (K-12042, Advansta).

## Supplementary information

Supplemental figures

tables

aj-checklist

## Data Availability

Raw and processed RNA-seq data described in the study are uploaded to the Gene Expression Omnibus (GEO) database under GSE221077 accession. Processed mESC metabolomics data and corresponding results are provided in Supplementary Table 5.

## References

[1] López-Otín C, Blasco MA, Partridge L, Serrano M, Kroemer G (2013). The Hallmarks of Aging. Cell.

[2] Mattson MP, Arumugam TV (2018). Hallmarks of brain aging: Adaptive and pathological modification by metabolic states. Cell Metab.

[3] Hernandez-Segura A, Nehme J, Demaria M (2018). Hallmarks of cellular senescence. Trends Cell Biol.

[4] Sahin E, DePinho RA (2012). Axis of ageing: telomeres, p53 and mitochondria. Nat Rev Mol Cell Biol.

[5] Peters R (2006). Ageing and the brain. Postgrad Med J..

[6] Blinkouskaya Y, Caçoilo A, Gollamudi T, Jalalian S, Weickenmeier J (2021). Brain aging mechanisms with mechanical manifestations. Mech Ageing Dev.

[7] Raz N, Lindenberger U, Rodrigue KM, Kennedy KM, Head D, Williamson A (2005). Regional brain changes in aging healthy adults: general trends, individual differences and modifiers. Cereb Cortex.

[8] Rine J, Strathern JN, Hicks JB, Herskowitz I (1979). A suppressor of mating-type locus mutations in Saccharomyces cerevisiae: evidence for and identification of cryptic mating-type loci. Genetics..

[9] Braunstein M, Rose AB, Holmes SG, Allis CD, Broach JR (1993). Transcriptional silencing in yeast is associated with reduced nucleosome acetylation. Genes Dev.

[10] Tanner KG, Landry J, Sternglanz R, Denu JM (2000). Silent information regulator 2 family of NAD- dependent histone/protein deacetylases generates a unique product, 1-O-acetyl-ADP-ribose. Proc Natl Acad Sci USA..

[11] Mostoslavsky R, Chua KF, Lombard DB, Pang WW, Fischer MR, Gellon L (2006). Genomic instability and aging-like phenotype in the absence of mammalian SIRT6. Cell..

[12] Van Meter M, Kashyap M, Rezazadeh S, Geneva AJ, Morello TD, Seluanov A (2014). SIRT6 represses LINE1 retrotransposons by ribosylating KAP1 but this repression fails with stress and age. Nat Commun.

[13] Onn L, Portillo M, Ilic S, Cleitman G, Stein D, Kaluski S (2020). SIRT6 is a DNA double-strand break sensor. Elife.

[14] Toiber D, Erdel F, Bouazoune K, Silberman DM, Zhong L, Mulligan P (2013). SIRT6 recruits SNF2H to DNA break sites, preventing genomic instability through chromatin remodeling. Mol Cell.

[15] Mao Z, Hine C, Tian X, Van Meter M, Au M, Vaidya A (2011). SIRT6 promotes DNA repair under stress by activating PARP1. Science..

[16] Michishita E, McCord RA, Berber E, Kioi M, Padilla-Nash H, Damian M (2008). SIRT6 is a histone H3 lysine 9 deacetylase that modulates telomeric chromatin. Nature..

[17] Roichman A, Elhanati S, Aon MA, Abramovich I, Di Francesco A, Shahar Y (2021). Restoration of energy homeostasis by SIRT6 extends healthy lifespan. Nat Commun.

[18] Kaluski S, Portillo M, Besnard A, Stein D, Einav M, Zhong L (2017). Neuroprotective Functions for the Histone Deacetylase SIRT6. Cell Rep.

[19] Li X, Liu L, Li T, Liu M, Wang Y, Ma H (2021). SIRT6 in senescence and aging-related cardiovascular diseases. Front Cell Dev Biol.

[20] Khan RI, Nirzhor SSR, Akter R (2018). A review of the recent advances made with SIRT6 and its implications on aging related processes, major human diseases, and possible therapeutic targets. Biomolecules..

[21] Garcia-Venzor A, Toiber D (2021). SIRT6 through the brain evolution, development, and aging. Front Aging Neurosci.

[22] Lee OH, Kim J, Kim JM, Lee H, Kim EH, Bae SK (2013). Decreased expression of sirtuin 6 is associated with release of high mobility group box-1 after cerebral ischemia. Biochem Biophys Res Commun.

[23] Portillo M, Eremenko E, Kaluski S, Garcia-Venzor A, Onn L, Stein D (2021). SIRT6-CBP-dependent nuclear Tau accumulation and its role in protein synthesis. Cell Rep.

[24] Stein D, Mizrahi A, Golova A, Saretzky A, Venzor AG, Slobodnik Z (2021). Aging and pathological aging signatures of the brain: through the focusing lens of SIRT6. Aging..

[25] Mecocci P, MacGarvey U, Kaufman AE, Koontz D, Shoffner JM, Wallace DC (1993). Oxidative damage to mitochondrial DNA shows marked age-dependent increases in human brain. Ann Neurol.

[26] Wallace DC (2005). A mitochondrial paradigm of metabolic and degenerative diseases, aging, and cancer: a dawn for evolutionary medicine. Annu Rev Genet.

[27] Park CB, Larsson NG (2011). Mitochondrial DNA mutations in disease and aging. J Cell Biol.

[28] Harman D (1956). Aging: a theory based on free radical and radiation chemistry. J Gerontol.

[29] Chakrabarti S, Munshi S, Banerjee K, Thakurta IG, Sinha M, Bagh MB (2011). Mitochondrial dysfunction during brain aging: Role of oxidative stress and modulation by antioxidant supplementation. Aging Dis.

[30] Raichle ME, Gusnard DA (2002). Appraising the brain’s energy budget. Proc Natl Acad Sci USA..

[31] Storozhuk MV, Ivanova SY, Balaban PM, Kostyuk PG (2005). Possible role of mitochondria in posttetanic potentiation of GABAergic synaptic transmission in rat neocortical cell cultures. Synapse..

[32] Motori E, Atanassov I, Kochan SMV, Folz-Donahue K, Sakthivelu V, Giavalisco P (2020). Neuronal metabolic rewiring promotes resilience to neurodegeneration caused by mitochondrial dysfunction. Sci Adv.

[33] Cunningham JT, Rodgers JT, Arlow DH, Vazquez F, Mootha VK, Puigserver P (2007). mTOR controls mitochondrial oxidative function through a YY1-PGC-1alpha transcriptional complex. Nature..

[34] Ansari A, Rahman MS, Saha SK, Saikot FK, Deep A, Kim KH (2017). Function of the SIRT3 mitochondrial deacetylase in cellular physiology, cancer, and neurodegenerative disease. Aging Cell.

[35] Dai SH, Chen T, Wang YH, Zhu J, Luo P, Rao W (2014). Sirt3 protects cortical neurons against oxidative stress via regulating mitochondrial Ca2+ and mitochondrial biogenesis. Int J Mol Sci.

[36] Rath S, Sharma R, Gupta R, Ast T, Chan C, Durham TJ (2021). MitoCarta3.0: an updated mitochondrial proteome now with sub-organelle localization and pathway annotations. Nucleic Acids Res.

[37] Sunkin SM, Ng L, Lau C, Dolbeare T, Gilbert TL, Thompson CL (2013). Allen Brain Atlas: an integrated spatio-temporal portal for exploring the central nervous system. Nucleic Acids Res..

[38] van de Ven RAH, Santos D, Haigis MC (2017). Mitochondrial Sirtuins and Molecular Mechanisms of Aging. Trends Mol Med.

[39] Zhang Y, Chen K, Sloan SA, Bennett ML, Scholze AR, O’Keeffe S (2014). An RNA-sequencing transcriptome and splicing database of Glia, neurons, and vascular cells of the cerebral cortex. J Neurosci.

[40] Mariottini C, Scartabelli T, Bongers G, Arrigucci S, Nosi D, Leurs R (2009). Activation of the histaminergic H3 receptor induces phosphorylation of the Akt/GSK-3 beta pathway in cultured cortical neurons and protects against neurotoxic insults. J Neurochem.

[41] Shao J, Yang X, Liu T, Zhang T, Xie QR, Xia W (2016). Autophagy induction by SIRT6 is involved in oxidative stress-induced neuronal damage. Protein Cell.

[42] Rayaprolu S, Bitarafan S, Santiago JV, Betarbet R, Sunna S, Cheng L (2022). Cell type-specific biotin labeling in vivo resolves regional neuronal and astrocyte proteomic differences in mouse brain. Nat Commun.

[43] Oberdoerffer P, Michan S, McVay M, Mostoslavsky R, Vann J, Park SK (2008). SIRT1 redistribution on chromatin promotes genomic stability but alters gene expression during aging. Cell.

[44] Wang X, Patel ND, Hui D, Pal R, Hafez MM, Sayed-Ahmed MM (2014). Gene expression patterns in the hippocampus during the development and aging of Glud1 (Glutamate Dehydrogenase 1) transgenic and wild type mice. BMC Neurosci.

[45] Chabi B, Ljubicic V, Menzies KJ, Huang JH, Saleem A, Hood DA (2008). Mitochondrial function and apoptotic susceptibility in aging skeletal muscle. Aging Cell.

[46] Jung ES, Choi H, Song H, Hwang YJ, Kim A, Ryu H (2016). p53-dependent SIRT6 expression protects Aá42-induced DNA damage. Sci Rep.

[47] Camacho-Pereira J, Tarragó MG, Chini CCS, Nin V, Escande C, Warner GM (2016). CD38 dictates age-related NAD decline and mitochondrial dysfunction through an SIRT3-dependent mechanism. Cell Metab.

[48] Mouchiroud L, Houtkooper RH, Moullan N, Katsyuba E, Ryu D, Cantó C (2013). The NAD+/sirtuin pathway modulates longevity through activation of mitochondrial UPR and FOXO signaling. Cell..

[49] Imai S, Guarente L (2014). NAD+ and sirtuins in aging and disease. Trends Cell Biol.

[50] Wood JG, Schwer B, Wickremesinghe PC, Hartnett DA, Burhenn L, Garcia M (2018). Sirt4 is a mitochondrial regulator of metabolism and lifespan in Drosophila melanogaster. Proc Natl Acad Sci USA..

[51] Min Z, Gao J, Yu Y (2018). The roles of mitochondrial SIRT4 in cellular metabolism. Front Endocrinol Lausanne.

[52] Kincaid B, Bossy-Wetzel E (2013). Forever young: SIRT3 a shield against mitochondrial meltdown, aging, and neurodegeneration. Front Aging Neurosci.

[53] Boxer LD, Renthal W, Greben AW, Whitwam T, Silberfeld A, Stroud H (2020). MeCP2 Represses the Rate of Transcriptional Initiation of Highly Methylated Long Genes. Mol Cell.

[54] Etchegaray JP, Chavez L, Huang Y, Ross KN, Choi J, Martinez-Pastor B (2015). The histone deacetylase SIRT6 controls embryonic stem cell fate via TET-mediated production of 5-hydroxymethylcytosine. Nat Cell Biol.

[55] Chen S, Zhou Y, Chen Y, Gu J (2018). fastp: an ultra-fast all-in-one FASTQ preprocessor. Bioinformatics..

[56] Ewels PA, Peltzer A, Fillinger S (2020). The nf-core framework for community-curated bioinformatics pipelines. Nat Biotechnol.

[57] Dobin A, Davis CA, Schlesinger F, Drenkow J, Zaleski C, Jha S (2013). STAR: ultrafast universal RNA-seq aligner. Bioinformatics..

[58] Patro R, Duggal G, Love MI, Irizarry RA, Kingsford C (2017). Salmon provides fast and bias-aware quantification of transcript expression. Nat Methods.

[59] Pagès H, Carlson M, Falcon S, Li N (2022). AnnotationDbi: Manipulation of SQLite-based annotations in Bioconductor. R package version 1.60.0, https://bioconductor.org/packages/AnnotationDbi.

[60] Rainer J (2017). *EnsDb.Mmusculus.v79: Ensembl based annotation package*. R package version 2.99.0.

[61] Love MI, Huber W, Anders S (2014). Moderated estimation of fold change and dispersion for RNA-seq data with DESeq2. Genome Biol.

[62] Yu G, Wang LG, Han Y, He QY (2012). clusterProfiler: an R package for comparing biological themes among gene clusters. OMICS..

[63] Yu G (2018). *enrichplot: Visualization of Functional Enrichment Result*. R package version 1.0.2, https://github.com/GuangchuangYu/enrichplot.

[64] Shannon P, Markiel A, Ozier O, Baliga NS, Wang JT, Ramage D (2003). Cytoscape: a software environment for integrated models of biomolecular interaction networks. Genome Res.

[65] Patil I (2021). Visualizations with statistical details: The ‘ggstatsplot’ approach. J Open Source Softw.

[66] Giavalisco P, Li Y, Matthes A, Eckhardt A, Hubberten HM, Hesse H (2011). Elemental formula annotation of polar and lipophilic metabolites using (13) C, (15) N and (34) S isotope labelling, in combination with high-resolution mass spectrometry: Isotope labelling for unbiased plant metabolomics. Plant J.

[67] Lapidot-Cohen T, Rosental L, Brotman Y (2020). Liquid chromatography-mass spectrometry (LC-MS)-based analysis for lipophilic compound profiling in plants. Curr Protoc Plant Biol.

[68] Pang Z, Chong J, Zhou G, de Lima Morais DA, Chang L, Barrette M (2021). MetaboAnalyst 5.0: narrowing the gap between raw spectra and functional insights. Nucleic Acids Res.

[69] Blighe, Kevin. 2018. “EnhancedVolcano: Publication-ready volcano plots with enhanced colouring and labeling.” https://github.com/kevinblighe.

[70] Kanehisa M, Sato Y, Kawashima M, Furumichi M, Tanabe M (2016). KEGG as a reference resource for gene and protein annotation. Nucleic Acids Res.

[71] Gu Z, Eils R, Schlesner M (2016). Complex heatmaps reveal patterns and correlations in multidimensional genomic data. Bioinformatics..

[72] Oki S, Ohta T, Shioi G, Hatanaka H, Ogasawara O, Okuda Y (2018). ChIP-Atlas: a data-mining suite powered by full integration of public ChIP-seq data. EMBO Rep.

[73] Yu G, Wang LG, He QY (2015). ChIPseeker: an R/Bioconductor package for ChIP peak annotation, comparison and visualization. Bioinformatics..

[74] Gel B, Serra E (2017). karyoploteR: an R/Bioconductor package to plot customizable genomes displaying arbitrary data. Bioinformatics..

[75] Barrett T, Wilhite SE, Ledoux P, Evangelista C, Kim IF, Tomashevsky M (2013). NCBI GEO: archive for functional genomics data sets-update. Nucleic Acids Res..

