# Supplementary material for: SIRT6 is a key regulator of mitochondrial function in the brain
Source: Cell Death Dis. 2023 Jan 18;14(1):35. doi: 10.1038/s41419-022-05542-w (PMC9849342; doi:10.1038/s41419-022-05542-w)
Supplement: Supplementary file 1 — Supplemental figures [file 41419_2022_5542_MOESM1_ESM.docx]

# Supplemental figures

**SIRT6 is a key regulator of mitochondrial function in the brain.**

Dmitrii Smirnov, Ekaterina Eremenko, Daniel Stein, Shai Kaluski, Weronika Jasinska, Claudia Consetino, Barbara Martinez-Pastor, Yariv Brotman, Raul Mostoslavsky, Ekaterina Khrameeva and Debra Toiber


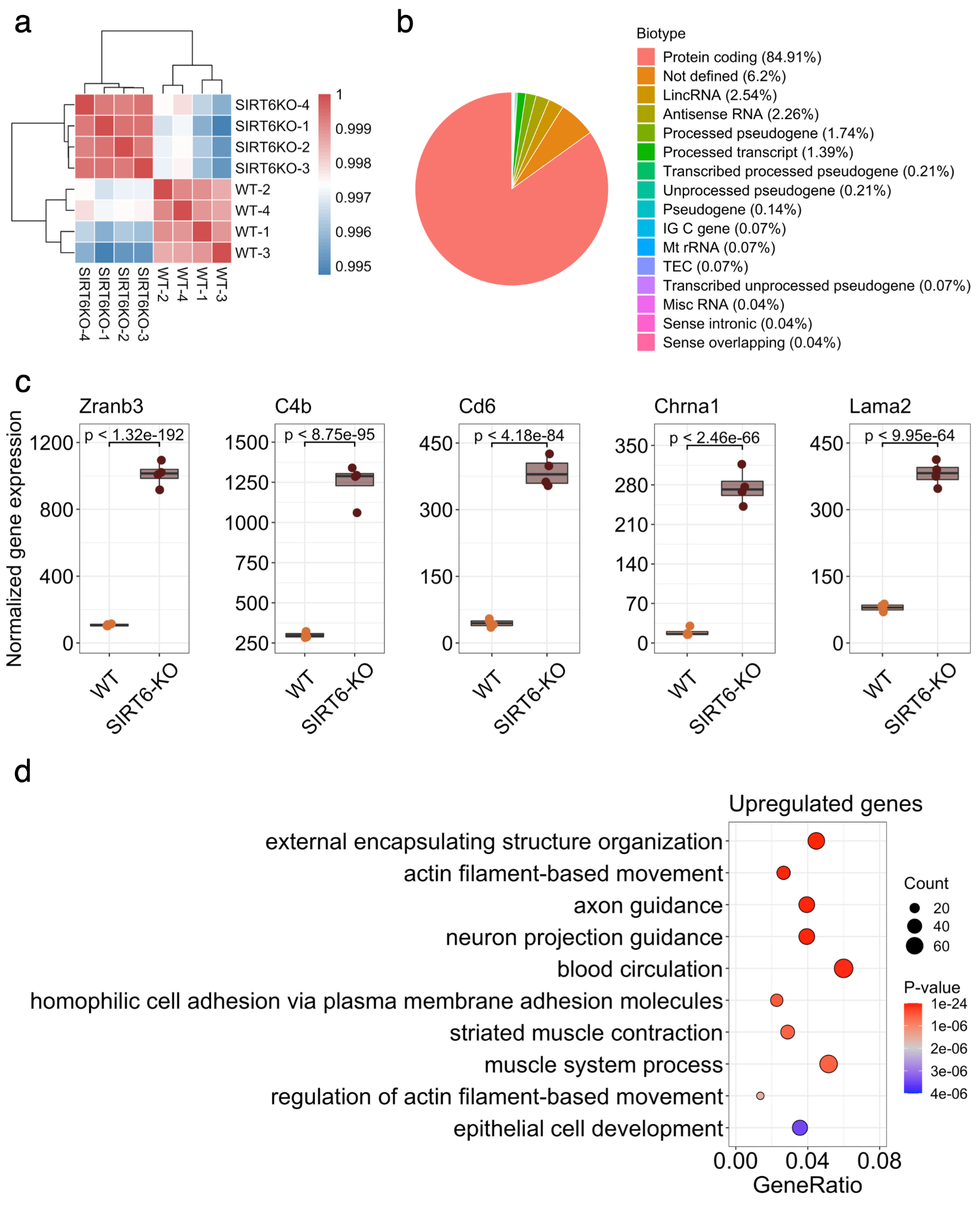


**Supplementary figure 1: Analysis of W.T. and brSIRT6-KO gene expression data (related to Fig. 1). (a)** Heatmap illustrating Pearson’s correlation between experimental samples. **(b)** Pie plot representing proportion of biotype annotations among DE genes. ‘IG C gene’ annotation denotes constant chain immunoglobulin genes, ‘TEC’ annotation describes predicted genes that require experimental validation. **(c)** Boxplots of the top 5 most differentially expressed genes in the analysis. Expression of WT samples are shown by orange points and expression of brSIRT6 are shown by brown points. **(d)** GO analysis showing top 10 enriched biological processes for upregulated genes. Each circle corresponds to the enriched GO term and varies in size according to the number of significant genes belonging to this term. Gene ratio represents the number of DE genes belonging to the enrichment categories divided by the total number of genes per category.


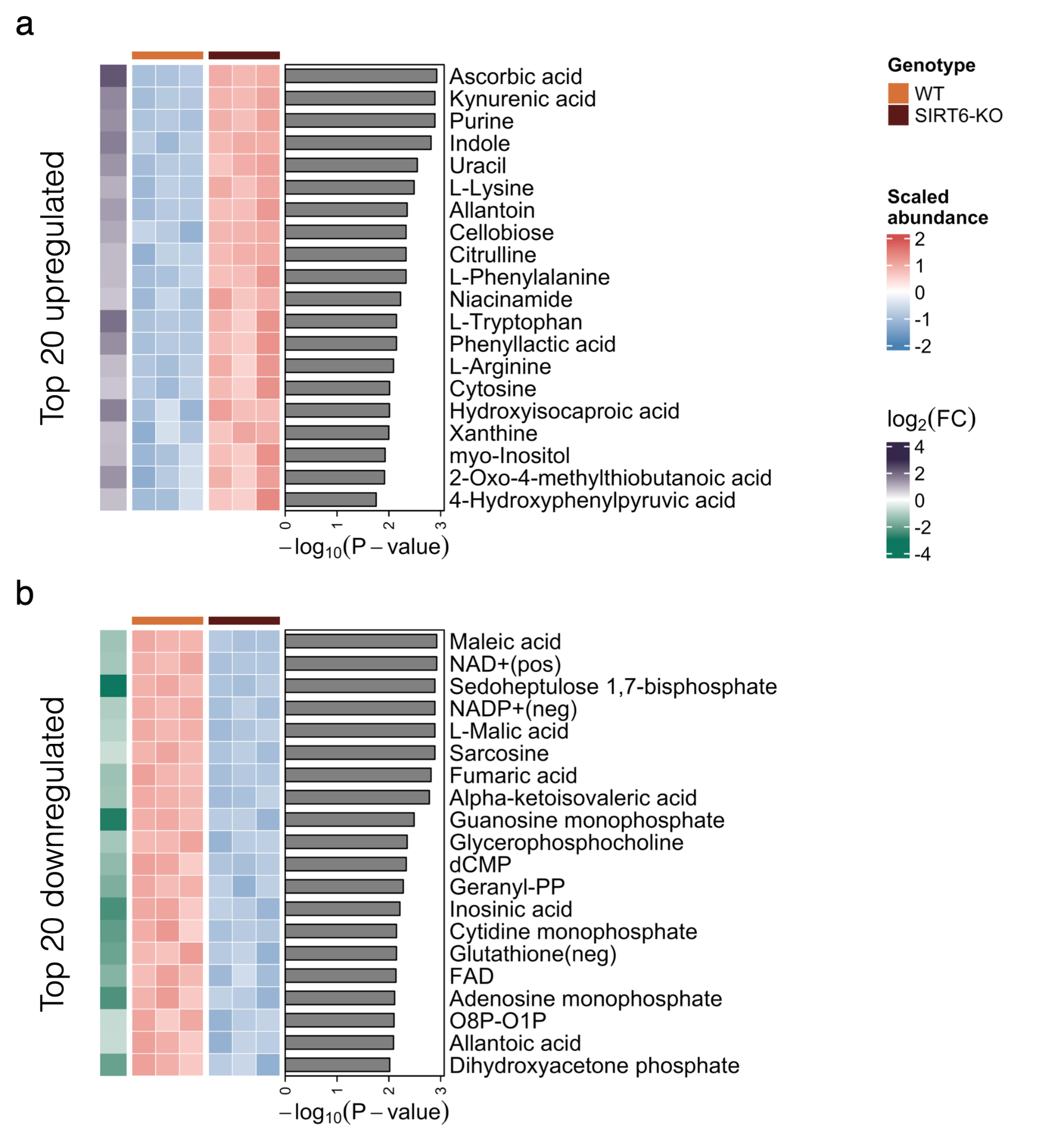


**Supplementary figure 2: mESC metabolomics (related to Fig. 2). (a,b)** Heatmaps showing abundances of the top 20 most significantly upregulated (panel a) and downregulated (panel b) in SIRT6-KO (brown) compared to WT (orange) metabolites. Row annotations on the left of the heatmaps represent log_2_ Fold Change values corresponding to the metabolites. Barplots on the right side of the heatmaps represent -log_10_ transformed FDR p-value of the metabolites.


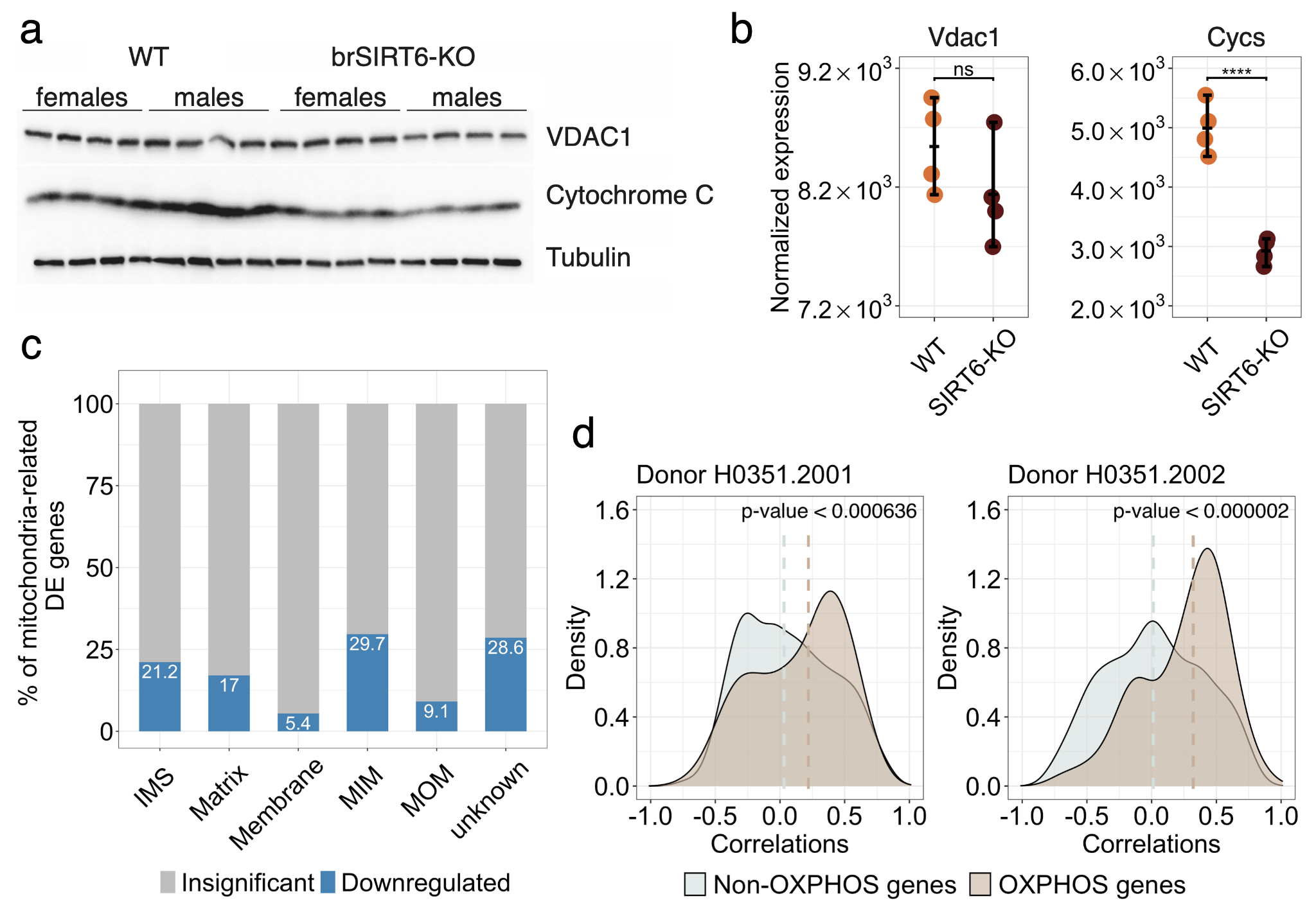


**Supplementary figure 3: SIRT6 regulates OXPHOS-related genes (related to Fig. 3). (a)** Western blot analysis of total brain extracts from W.T. (n=8) and brSIRT6-KO brains (n=8). The total brain fraction was prepared as described in Materials and Methods. Membrane blots were incubated with antibodies against Cytochrome C, Tubulin and VDAC. **(b)** *Vdac1* and *Cycs* expression levels in W.T. and SIRT6-KO RNA-seq profiles. **(c)** The percentage of significant (blue bars) and insignificant (gray bars) genes across mitochondrial compartments. 'IMS' denotes intermembrane space, 'MIM' denotes mitochondrial inner membrane, and 'MOM' corresponds to the mitochondrial outer membrane. **(d)** Spearman’s correlation value distributions for SIRT6 with OXPHOS-related (brown shapes) and other mitochondria-related genes (blue shapes) in the Allen Brain Atlas RNA-seq datasets of two donors (H0351.2001, H0351.2002). Brown and blue dashed lines correspond to medians of correlation distributions for OXPHOS and non-OXPHOS genes, respectively. Statistical significance for SIRT6 correlation with OXPHOS-related genes is calculated via permutation test.


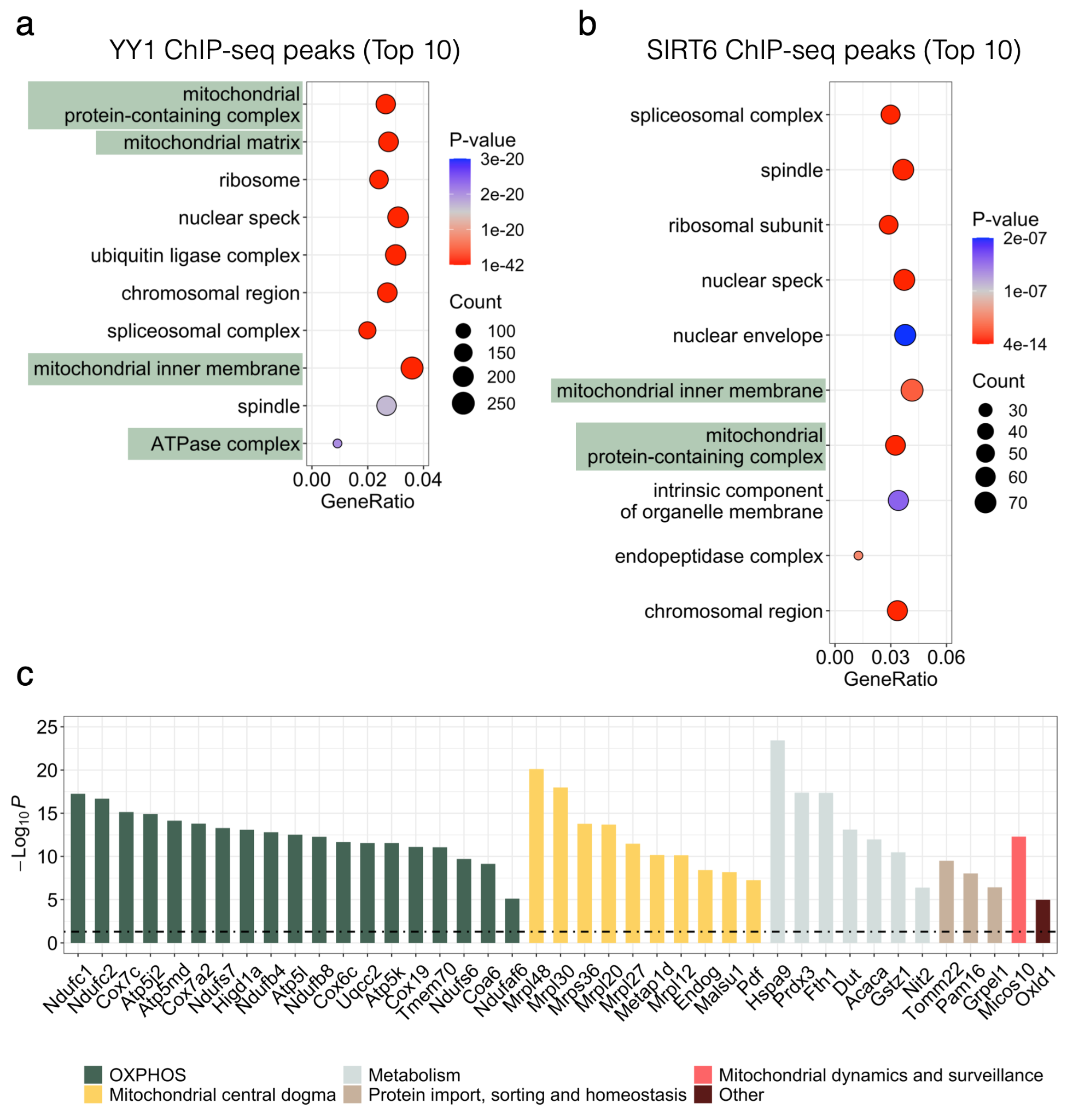


**Supplementary figure 4: Analysis of public YY1 and SIRT6 ChIP-seq datasets (related to Fig. 4).** **(a-b)** Top 10 significant cellular component terms from GO ontology analysis of genes associated with YY1 (panel a) and SIRT6 (panel b) peaks. Mitochondria-related cell compartments are marked by green. **(c)** Barplot showing the expression change magnitudes of genes overlapped between all the datasets presented in Fig. 4d. Bars are colored according to the cellular function of corresponding genes. Black dashed denotes significance cut off for -log_10_ FDR p-value.


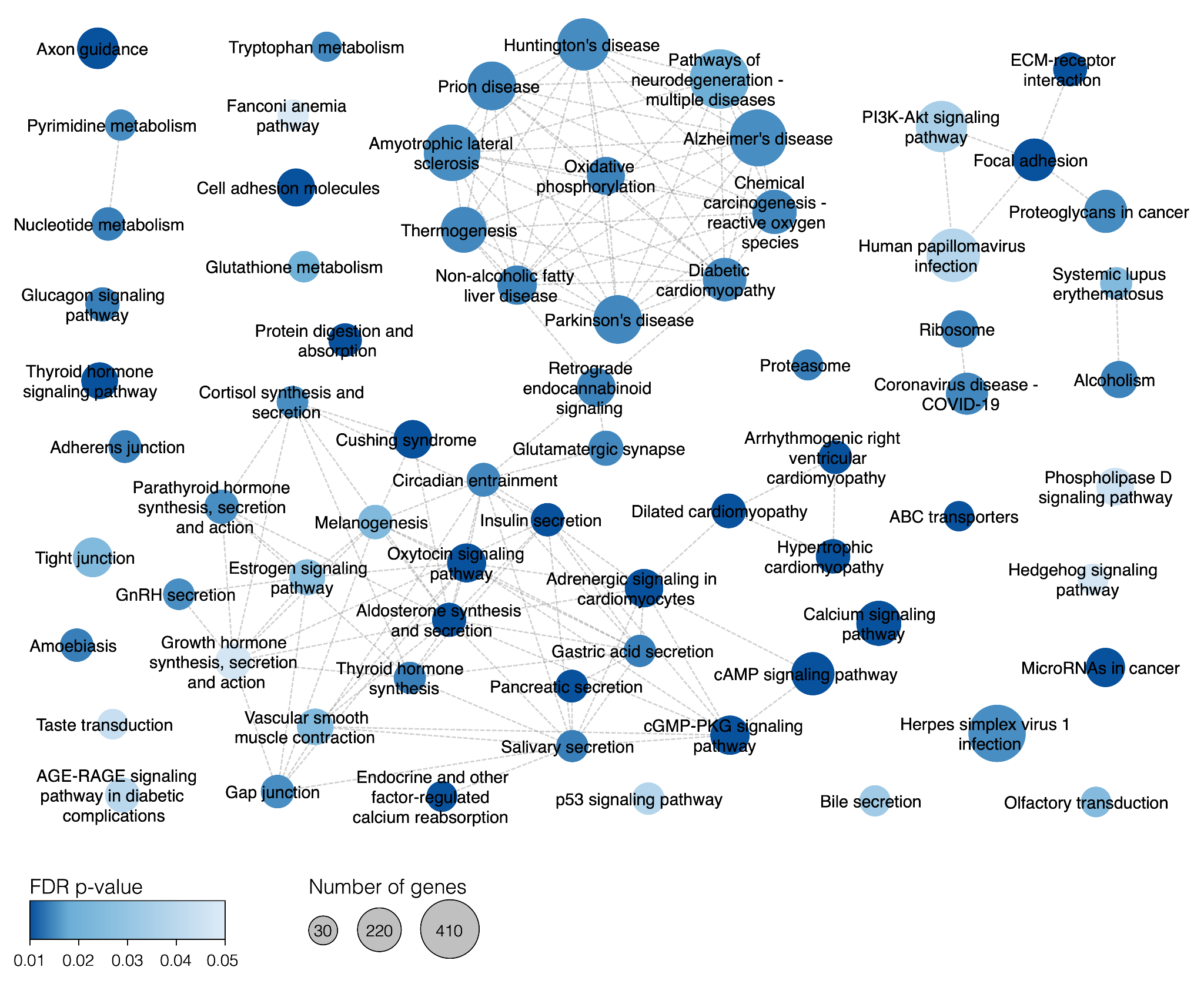


**Supplementary figure 5: Full network of enriched KEGG pathways (related to Fig. 5).** Each circle represents an enriched pathway in GSEA analysis and is colored according to the FDR p-value. The size of circles corresponds to the number of detected genes related to the particular pathway.
